# Associations between environmental covariates and temporal changes in malaria incidence in high transmission settings of Uganda: a distributed lag nonlinear analysis

**DOI:** 10.1186/s12889-021-11949-5

**Published:** 2021-10-30

**Authors:** Jaffer Okiring, Isobel Routledge, Adrienne Epstein, Jane F. Namuganga, Emmanuel V. Kamya, Gloria Odei Obeng-Amoako, Catherine Maiteki Sebuguzi, Damian Rutazaana, Joan N. Kalyango, Moses R. Kamya, Grant Dorsey, Ronald Wesonga, Steven M. Kiwuwa, Joaniter I. Nankabirwa

**Affiliations:** 1 Clinical Epidemiology Unit, School of Medicine, Makerere University College of Health Sciences, Kampala, Uganda; 2 Infectious Diseases Research Collaboration, 2C Nakasero Hill Road, Kampala, Uganda; 3 Department of Epidemiology and Biostatistics, University of California, San Francisco, USA; 4 National Malaria Control Division, Ministry of Health, Kampala, Uganda; 5 School of Medicine, Makerere University College of Health Sciences, Kampala, Uganda; 6 Department of Medicine, University of California, San Francisco, USA; 7 Department of Statistics, College of Science, Sultan Qaboos University, Muscat, Oman; 8 Department of Child Health and Development Centre, School of Medicine, Makerere University College of Health Sciences, Kampala, Uganda

**Keywords:** Environmental, Covariates, Temporal, Effect, Malaria, Incidence, DLNM

## Abstract

**Background:**

Environmental factors such as temperature, rainfall, and vegetation cover play a critical role in malaria transmission. However, quantifying the relationships between environmental factors and measures of disease burden relevant for public health can be complex as effects are often non-linear and subject to temporal lags between when changes in environmental factors lead to changes in malaria incidence. The study investigated the effect of environmental covariates on malaria incidence in high transmission settings of Uganda.

**Methods:**

This study leveraged data from seven malaria reference centres (MRCs) located in high transmission settings of Uganda over a 24-month period. Estimates of monthly malaria incidence (MI) were derived from MRCs’ catchment areas. Environmental data including monthly temperature, rainfall, and normalized difference vegetation index (NDVI) were obtained from remote sensing sources. A distributed lag nonlinear model was used to investigate the effect of environmental covariates on malaria incidence.

**Results:**

Overall, the median (range) monthly temperature was 30 °C (26–47), rainfall 133.0 mm (3.0–247), NDVI 0.66 (0.24–0.80) and MI was 790 per 1000 person-years (73–3973). Temperature of 35 °C was significantly associated with malaria incidence compared to the median observed temperature (30 °C) at month lag 2 (IRR: 2.00, 95% CI: 1.42–2.83) and the increased cumulative IRR of malaria at month lags 1–4, with the highest cumulative IRR of 8.16 (95% CI: 3.41–20.26) at lag-month 4. Rainfall of 200 mm significantly increased IRR of malaria compared to the median observed rainfall (133 mm) at lag-month 0 (IRR: 1.24, 95% CI: 1.01–1.52) and the increased cumulative IRR of malaria at month lags 1–4, with the highest cumulative IRR of 1.99(95% CI: 1.22–2.27) at lag-month 4. Average NVDI of 0.72 significantly increased the cumulative IRR of malaria compared to the median observed NDVI (0.66) at month lags 2–4, with the highest cumulative IRR of 1.57(95% CI: 1.09–2.25) at lag-month 4.

**Conclusions:**

In high-malaria transmission settings, high values of environmental covariates were associated with increased cumulative IRR of malaria, with IRR peaks at variable lag times. The complex associations identified are valuable for designing strategies for early warning, prevention, and control of seasonal malaria surges and epidemics.

**Supplementary Information:**

The online version contains supplementary material available at 10.1186/s12889-021-11949-5.

## Background

Environmental covariates such as temperature, vegetation, and rainfall play a major role in malaria transmission [1–3], by changing the vector populations which often lead to changes in malaria burden and yet the quantitative relationships between changes in these covariates and malaria incidence are not well characterized in many settings especially in sub Saharan Africa. Several factors complicate the characterization of these relationships. Firstly, the effect of environmental covariates on mosquito and parasite populations may not be linear. For instance, moderate increase in rainfall leads to increased humidity which prolongs adult longevity of the mosquitoes and a surge in their population while heavy rainfall reduces the populations by washing away the mosquito larvae [4]. Similarly, temperature is a crucial factor in the vector life-cycle. For instance, a rise in temperature may also increase the blood meals taken and eggs laid by the mosquito, increasing mosquito-population density affecting transmission. Lower temperatures, especially below 20 °C, and too high temperatures may hamper the completion of mosquito growth cycle [5, 6]. Vegetation may provide an outdoor resting habitant or shelter for mosquitoes from extreme conditions unfavourable for mosquito-population growth. Many studies have reported associations between changes in malaria burden and patterns of environmental factors [7–13]. However, the associations reported vary between settings. For example, a study from South Africa found that an increase in temperature significantly raised malaria infections [12], while another in Ethiopia showed a negative correlation [13].

Environmental covariates may also show effects that are delayed in time, requiring examination of the temporal dimension of the exposure–lag-response relationship. Most studies on the relationships between these covariates and the malaria burden have relied on specific time lag, ignoring the cumulative effect of the environmental covariates which may last for a period longer than the current time [7, 14, 15]. From the biological perspective, different periods including time for mosquito to develop, period of parasites within the mosquito, and incubation period of the parasites within human body makes the assumption of a specific time lag unrealistic, as the observed effect of the environmental covariates in a given lag may be a cumulative effect from the preceding lags. Additionally, the occurrence of extreme environmental conditions in the recent past such as prolonged rainfall seasons may have an impact on malaria burden which is not yet clear.

Climate change has had great impacts on infectious diseases, with shifts in malaria transmission areas reported [16, 17], as may be reflected in changes of malaria burden provided through surveillance data. Routine malaria surveillance focuses on measures of disease (rather than entomological measures) and measures of disease are of greatest relevance from a public health perspective. Recently Uganda has experienced extreme environmental conditions amidst a setting where malaria is already endemic in almost 95% of the country [18], and yet there is limited data on the quantitative relationship between these covariates and malaria. Uganda Malaria Surveillance Project (UMSP) in collaboration with National Malaria Control Division (NMCD) have established an enhanced health facility-based malaria surveillance system at 70 public health facilities across the country referred to as the Malaria Reference Centers (MRCs) [19]. At these MRCs, individual patient level data are collected and resources provided to maximize laboratory testing of all patients with suspected malaria. Data on village of residence of the patients is captured and catchment areas around the MRCs identified, allowing for the generation of estimates of malaria incidence (Program for resistance, immunology, surveillance, and modelling of malaria (prism) : Implementation Project Pilot Study, Unpublished). In this study, the effect of environmental variability in rainfall, temperature and vegetation on malaria incidence in Uganda is quantified by investigating exposure-lag-response effects. Quantifying these relationships is a key step in producing useful systems to predict malaria incidence in the region and plan for effective preventive strategies and sustainable long-term malaria programming in the control of malaria burden.

## Methods

### Study setting

This study leveraged data from UMSP derived from sentinel surveillance in level III and IV public outpatient facilities that generally see between 1000 and 3000 outpatients per month and have functioning laboratories. These facilities provide care free of charge, including diagnostic testing and medications. Full description of the MRCs and the data captured has been published else where [20]. This study included data from seven of the 70 MRCs. MRCs were included if they met the following criteria: 1) location in a high malaria burden area where indoor residual spraying of insecticide (IRS) was not being implemented, 2) had malaria incidence estimate data for the period between January 2019 to December 2020 available. MRCs included in the analysis were Aduku health centre IV in Kwania District, Lobule health centre III in Koboko District, Awach health centre IV in Gulu District, Lalogi health centre IV in Omoro District, Patongo health centre IV in Agago District, Padibe health centre IV in Lamwo District, Namokora health centre IV in Kitgum District. The location of these MRCs in Uganda is shown in supplementary file Fig. S1.

### Environmental variables

Average monthly environmental data for the period of January 2019–December 2020 were processed from remote sensing sources. Data processed by remote sensing included temperature (defined as day time land surface temperature measured in degrees Celsius), Normalized Difference Vegetation Index (NDVI) defined as a dimensionless index used to measure neighborhood greenness [21], and rainfall. Rainfall data was collected from climate hazards group infrared precipitation with station data (CHIRPS) database and was measured in millimeters. CHIRPS incorporate 0.05° resolution satellite imagery with in-situ station data to create gridded rainfall time series for trend analysis and seasonal drought monitoring [22]. Temperature and NDVI data was obtained from moderate resolution imaging spectro-radiometer (MODIS) aboard the National Aeronautics and Space Administration (NASA) satellites [23]. Global MODIS data are provided every month at 1-km spatial resolution as a gridded level-3 product in the sinusoidal projection and were gap-filled to correct for cloud cover using a random forest model with interpolated values, elevation, and time [24]. Satellite environmental covariates were preferred over nationally available estimates since they had been shown to have an even spatial distribution [25], and were available at a low administrative level such as a village, enabling derivation of health facility catchment area-specific estimates. The downloaded raster files were transferred into quantum geographical Information system (QGIS) software and village corresponding environmental covariates’ centroid values were extracted using Point Sampling tool. To give MRC specific estimates of environmental covariate in a given month, the centroid values corresponding to the villages that form the catchment area were averaged. Low values of each covariate (temperature, rainfall, and vegetation cover) included any value below the observed median while high values were those greater than the median for each respective environmental covariate.

### Outcome

The outcome was monthly malaria incidence defined as total cases of malaria within a given health facility catchment area divided by the population of the catchment area. Catchment areas were defined as villages where the MRC was located and adjacent villages with similar malaria incidence to the village where the MRC is located. Details of how the catchment areas were estimated are published else where [20]. A given catchment area included 1–5 villages. The village level population estimates for each catchment area were obtained from the AfriPop database and included a fixed population growth function of 0.0029 per unit time [26].

### Statistical analysis

Cumulative data for the characteristics of the study populations over the 24-month observation period (January 2019 – December 2020) were summarized and presented as monthly medians with corresponding ranges. A cross-correlation analysis was performed to ascertain the magnitude and direction of time-lagged relationships between environmental covariates and malaria incidence, and estimate the optimal lags. Optimal lags were defined as the month corresponding to the highest significant correlation coefficient. The Granger causality Wald test was performed to determine the likely effect of lagged environmental factors on the variability of malaria incidence. The distributed lag nonlinear model (DLNM) was used to investigate non-linear and lagged (specific and cumulative) effects of environmental covariates on the malaria incidence.

The DLNM is a modeling framework used to investigate associations with potentially non-linear and delayed effects on time-series data [27]. This methodology is based on the definition of a cross-basis, which is a function expressed by the combination of two sets of basic functions that specify the relationships in the dimension of predictor and time lags, respectively. Second order natural cubic spline for environmental factors that generated a basis matrix of polynomials was used for non-linear effect and lag effect. The more flexible lag effects at shorter delays were obtained by placing spline knots at equal intervals in the range of environmental variables and in the lag scale. Seasonality of malaria transmission was controlled by including four degrees of freedom per year in the model, representing the bimodal malaria peak seasons in Uganda [28]. A health facility-specific random variable was added to the model to control for unmeasured differences between the facilities. The model was selected on the basis of the Quasi-Akaike Information Criterion (QAIC). The median value for each variable was defined as the baseline reference for calculating the IRR of the separate effect (in a specific lag-month) and cumulative effect (in all months preceding a specific lag-month) on the malaria incidence. All the analyses were performed using R software version 3.6.0 with “dlnm” and “lme4” packages. Statistical significance was determined using confidence intervals that do not include the RR of the null hypothesis of 1.0.

A thousand simulations were run to rule out the possibility of the effects being solely an influence of multi-collinearity between temperature, rainfall, and vegetation cover using the methodology proposed by Jose Barrera-G’omez and Xavier Basagana in the “Collin” package in R [29]. The results are presented in the supplementary file Fig. S2 and the findings suggest the possibility of other explanations for this result than multi-collinearity.

## Results

### Summary data on longitudinal measures of malaria incidence and environmental covariates in high transmission settings of Uganda

Over the 24-month study period, the overall median monthly malaria incidence was 790 (range 73–3973) cases per 1000 person years (PY), with the catchment area around Patongo health centre having the highest incidence at 1272 (176–3973) cases per 1000 PY, and area around Namokora health centre having the lowest incidence at 337.5 (73–1238) cases per 1000 PY. The overall median temperature was 30.0 °C with Padibe and Namokora health centre recording the highest temperatures (30.5 °C) and Lobule health centre recording the lowest at 28.0 °C. The median monthly rainfall was 133.0 mm with highest estimates around Lalogi health centre (148.5 mm, 8-214 mm) and lowest around Padibe health centre (111.5 mm, 6-227 mm). NDVI was highest at Lobule health centre (0.74) and lowest at Patongo health centre (0.61) with the median across all-sites estimated at 0.66. Table 1 provides the details of the longitudinal measures of environmental variables at the study sites between January 2019 and December 2020.

**Table 1:** Summary data on longitudinal measures of Environmental variables in high transmission settings of Uganda 2019–2020

Site	Monthly median (range)		
	Temperature (degrees Celsius)	Rain fall (mm)	NDVI (index)
Aduku HC	29.00 (27.00–40.00)	142.00 (8.00–247.00)	0.68 (0.35–0.75)
Awach HC	29.00 (27.00–42.00)	138.50 (7.00–232.00)	0.68 (0.41–0.74)
Lalogi HC	28.50 (26.00–40.00)	148.50 (8.00–214.00)	0.72 (0.39–0.77)
Patongo HC	30.00 (28.00–47.00)	129.50 (3.00–223.00)	0.61 (0.25–0.69)
Padibe HC	30.50 (27.00–44.00)	111.50 (6.00–227.00)	0.62 (0.28–0.72)
Namokora HC	30.50 (27.00–47.00)	112.00 (4.00–226.00)	0.62 (0.24–0.75)
Lobule HC	28.00 (26.00–41.00)	122.00 (15.00–231.00)	0.73 (0.33–0.80)
All-sites combined	30.00 (26.00–47.00)	133.00 (3.00–247.00)	0.66 (0.24–0.80)

*NDVI* Normalized difference vegetation index

### Temporal trend and seasonality of malaria incidence and environmental covariates

Malaria incidence across all-sites was highest in June 2019 (1344.5 cases per 1000 PY, 713–2922) and lowest in April 2019 (239.5 cases per 1000 PY, 103–1128) with seasonal peak in incidence observed from April to September 2019 and accounting for 28.9% of the observed malaria incidence. Temporal changes in monthly malaria incidence over the 24-month observation period by MRC are presented in the supplementary file Fig. S3. Correlation analysis revealed a positive relationship between temperature and malaria incidence at month lag 4 (0.452), and a negative correlation for both rainfall (− 0.160) and NDVI (− 0.454) with malaria incidence at month lag 4. Across MRCs, the correlation coefficients for temperature with malaria incidence were negative at month lag 1 and positive at month lag 4. This pattern was reversed for both rainfall and NDVI at month lags 1 and 4. In addition, the optimal lags for the correlations between environmental covariates and malaria incidence varied by site (Table 2). The results of the Granger causality tests indicated that the temporal distribution of malaria incidence was strongly affected by temperature, rainfall, and NDVI among all-sites combined (Table 3).

**Table 2:** Cross correlation coefficients between environment factors and the malaria incidence among high transmission settings of Uganda 2019–2020

Site	Environmental variables								
	Temperature			Rain fall			NDVI		
	Optimal	Lag 1	Lag 4	optimal	Lag 1	Lag 4	optimal	Lag 1	Lag 4
Aduku HC	−0.417 (−1)*	− 0.417	0.366	0.511 (− 1)*	0.511	− 0.113	0.620 (− 1)*	0.620	− 0.299
Awach HC	0.496 (−4)*	− 0.301	0.496	0.416 (−1)*	0.416	−0.335	0.471 (−1)*	0.471	−0.617
Lalogi HC	0.613 (−4)*	−0.274	0.613	0.458 (−1)*	0.458	−0.510	−0.717 (−4)*	0.243	− 0.717
Patongo HC	0.481 (−4)*	−0.424	0.481	0.485 (−1)*	0.485	−0.205	0.453 (−1)*	0.453	−0.559
Padibe HC	−0.566 (−1)*	− 0.566	0.477	0.731 (−1)*	0.731	−0.120	0.426 (−1)*	0.426	−0.555
Namokora HC	−0.668 (−1)*	− 0.668	0.411	0.651 (−1)*	0.617	−0.009	0.609 (−1)*	0.609	−0.460
Lobule HC	−0.466 (−1)*	− 0.466	0.334	0.526 (−1)*	0.551	−0.195	0.534 (−1)*	0.525	−0.336
All-sites combined	0.452 (−4)*	−0.302	0.452	0.403 (−1)*	0.403	−0.160	−0.454 (−4)*	0.275	− 0.454

*NDVI* Normalized difference vegetation index

**Table 3:** Granger casuality tests for environmental factors (variables) and monthly malaria incidence in high transmission settings of Uganda 2019–2020

Site	Environmental variables		
	Temperature F-statistics (*p* value)	Rain fall F-statistics (*p* value)	NDVI F-statistics (*p* value)
Aduku HC	0.0004 (0.9839)	4.3100 (0.051)	1.2388 (0.2789)
Awach HC	0.8251 (0.536)	3.2346 (0.0872)	3.8258 (0.0646)
Lalogi HC	1.2804 (0.3356)	1.8236 (0.1920)	4.9558 (0.0157)
Patongo HC	1.1143 (0.3982)	3.6732 (0.0697)	0.8423 (0.3697)
Padibe HC	0.0732 (0.7895)	8.8014 (0.0076)	0.7569 (0.3946)
Namokora HC	1.8881 (0.1846)	2.0669 (0.1660)	0.6985 (0.4131)
Lobule HC	3.2905 (0.0847)	6.0919 (0.0227)	5.3309 (0.0318)
All-sites combined	7.9999 (< 0.0001)^a^	8.9646 (0.0032)^a^	8.4206 (< 0.0001)^a^

^a^Temporal distribution of malaria incidence is strongly affected by the respective enviromental factors

*NDVI* Normalized difference vegetation index

### Non-linear and lagged effects of environmental covariates on malaria incidence

#### Temperature

**Fig. 1:**
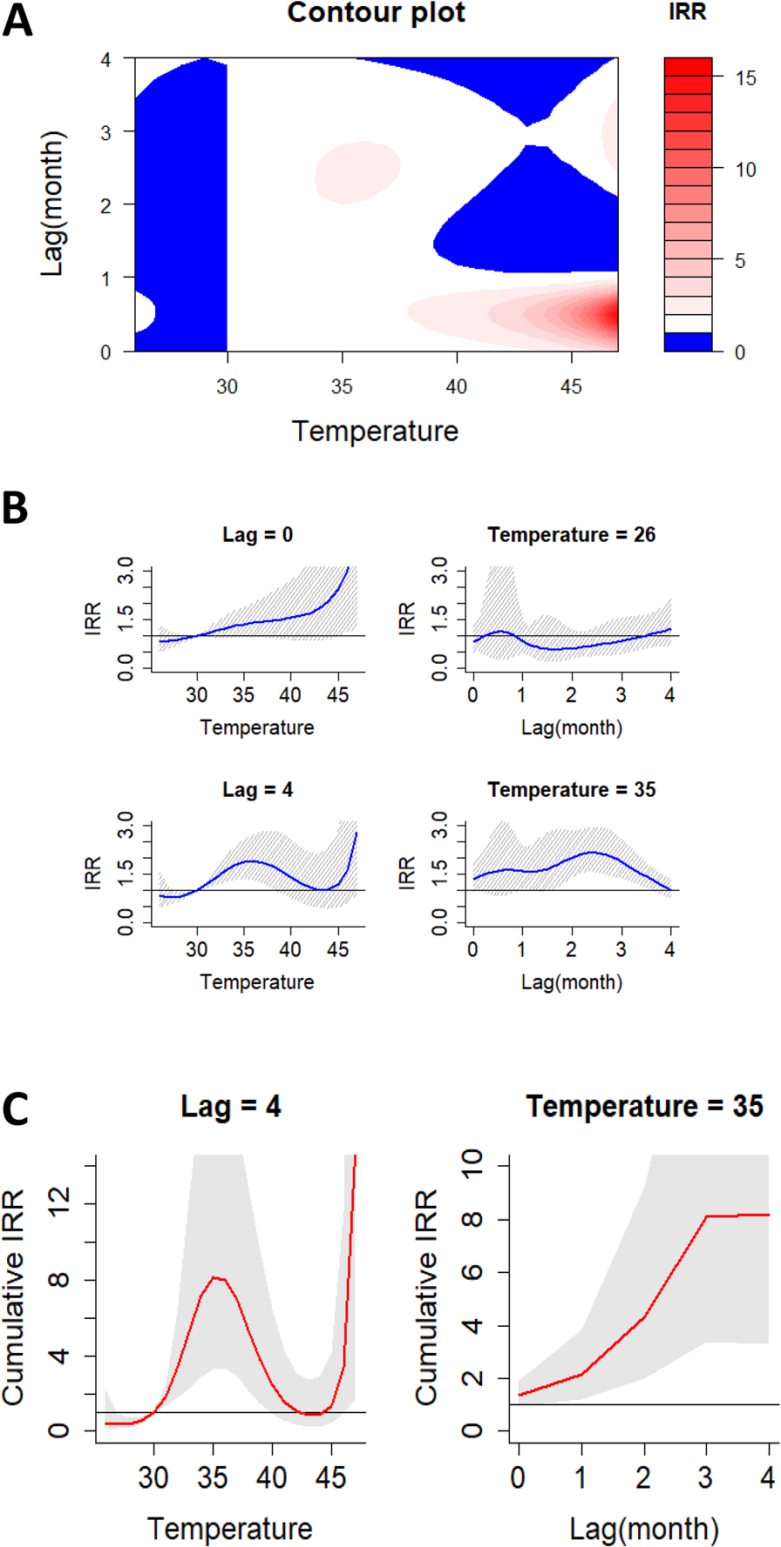
**a** Contour plots of the combined effect of time lags and Temperature on the incidence risk ratio of malaria. **b** Effect of specific Temperature and time lags on the incidence risk ratio of malaria. The blue lines are the mean relative risks, and the gray lines are 95% CI. **c** Effects of specific Temperature and time lags on the cumulative incidence risk ratio of malaria. The red lines are the mean incidence risk ratio, and the gray areas are 95% CI

With all sites combined; the incidence rate ratio (IRR) of malaria increased at month lags 0–1 for temperature approximately 45–47 °C compared to the median observed temperature (30.0 °C). Complete summary of the non-linear relationship between monthly temperature and malaria incidence over a four-month period is revealed in part a of Fig. 1. The separate effects of different temperatures and 2 month lags (0 and 4 months) on the IRR together with the 95% confidence intervals are provided in part b of Fig. 1. Temperature increased the IRR steadily at month lag 0 and increased the IRR to a peak at month lag 4. At low temperature, the IRR increased to 1.22 (95% CI, 0.68–2.16) at approximately 26.0 °C in month lag 4 as compared to the median observed temperature (30.0 °C). At temperature of approximately 35 °C, the IRR increased significantly to 2.00 (95% CI, 1.42–2.83) in month lag 2 compared to the median observed temperature (30.0 °C) (Table 4). The effect of temperature on the cumulative IRR of malaria is shown in part c of Fig. 1. Temperature of approximately 35 °C increased the cumulative IRR significantly at month lags 1–4 compared to the median observed temperature (30.0 °C) and the IRR of 8.16 (95% CI, 3.41–20.26) was the highest at month lag 4 (Table 4).

**Table 4:** DLNM model results for separate and cumulative effects of environmental variables on the RR of malaria burden in high transmission settings of Uganda

Effect type	Specification	Statistic	Variable		
			Temperature	Rainfall	NDVI
Separate effect	Low	Variable value	26	3	0.24
		Peak month	4	4	2
		IRR at peak month	1.22 (0.68–2.16)	4.05 (1.40–11.54)^a^	1.80 (0.35–9.43)
	High	Variable value	35	200	0.72
		Peak month	2	0	2
		IRR at peak month	2.00 (1.42–2.83)^a^	1.24 (1.01–1.52)^a^	1.31 (1.04–1.65)^a^
Cumulative effect	Month lag 1	Variable value	26	3	0.24
		IRR	0.69 (0.31–1.62)	2.52 (0.72–8.56)	0.44 (0.08–2.36)
		Variable value	35	200	0.72
		IRR	2.19 (1.21–3.89)^a^	1.50 (1.12–2.00)^a^	1.09 (0.87–1.38)
	Month lag 2	Variable value	26	3	0.24
		IRR	0.43 (0.14–1.42)	3.16 (0.57–17.41)	0.79 (0.13–4.78)
		Variable value	35	200	0.72
		IRR	4.39 (2.09–9.21)^a^	1.87 (1.31–2.69)^a^	1.42 (1.06–1.89)^a^
	Month lag 3	Variable value	26	3	0.24
		IRR	0.36 (0.10–1.55)	6.73 (0.64–68.29)	0.54 (0.06–4.68)
		Variable value	35	200	0.72
		IRR	8.08 (3.41–20.26)^a^	1.95 (1.28–2.97)^a^	1.42 (1.04–1.95)^a^
	Month lag 4	Variable value	26	3	0.24
		IRR	0.44 (0.10–2.19)	26.70 (1.82–397.00)^a^	0.83 (0.09–7.18)
		Variable value	35	200	0.72
		IRR	8.16 (3.41–20.26)^a^	1.99 (1.22–2.27)^a^	1.57 (1.09–2.25)^a^

Peak month is the month corresponding to the highest IRR of malaria

^a^statistically significant

*IRR* Incidence risk ratio

*NDVI* Normalized difference vegetation index

#### Rainfall

**Fig. 2:**
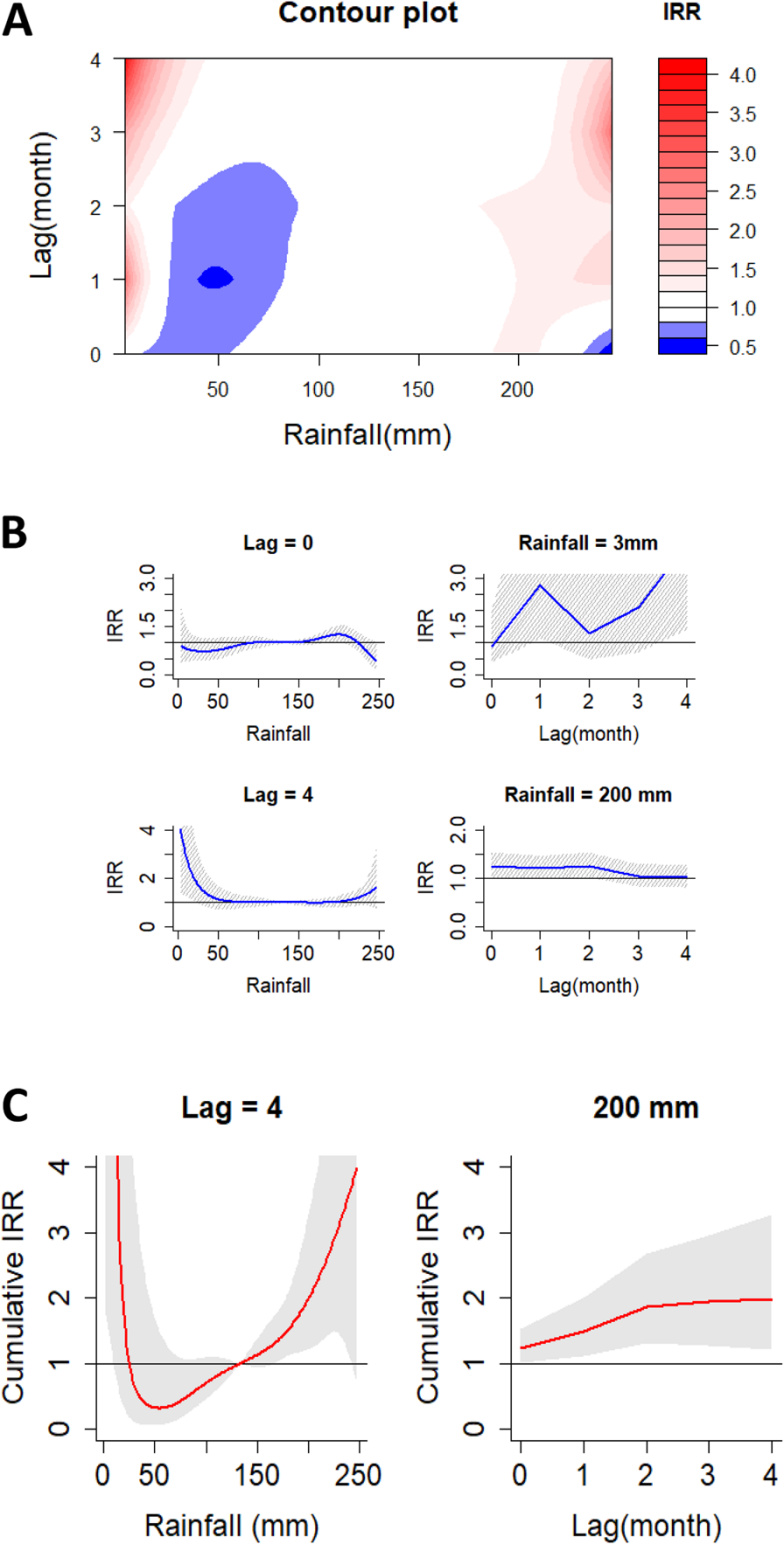
**a** Contour plots of the combined effect of time lags and rainfall amounts on the incidence risk ratio of malaria. **b** Effect of specific rainfall amounts and time lags on the incidence risk ratio of malaria. The blue lines are the mean incidence risk ratio, and the gray lines are 95% CI. **c** Effects of specific rainfall amounts and time lags on the cumulative incidence risk ratio of malaria. The red lines are the mean incidence risk ratio, and the gray areas are 95% CI

A summary of the non-linear relationship between monthly rainfall and malaria incidence over a four-month period is revealed in part a of Fig. 2. The IRR of malaria incidence increased at low rainfall in lag-month 4 and for rainfall approximately above 200 mm in month lags 1–4 compared to the median observed rainfall (133 mm). The separate effects of different rainfall values and two-month lags (0 and 4 months) on the IRR together with the 95% confidence intervals are provided in part b of Fig. 2. Increase in rainfall increased the IRR steadily to a peak at approximately 200 mm. While at month lag 4, increase in rainfall reduced the IRR drastically for values below 50 mm and flattened at IRR of 1.0 for values approximately 50-200 m. Overall at low rainfall, the IRR increased significantly to 4.05 (95% CI, 1.40–11.54) at approximately 3 mm in month lag 4 compared to the median observed rainfall (133 mm). At high rainfall, the IRR increased significantly to 1.24 (95% CI, 1.01–1.52) as compared to the median observed rainfall (133 mm) at approximately 200 mm in month lag 0 (Table 4). The effect of rainfall on the cumulative IRR of malaria is shown in part c of Fig. 2. Rainfall of approximately 200 mm increased the cumulative IRR from month lags 1–4 compared to the median observed rainfall (133 mm) and the RR of 1.99 (95% CI, 1.22–2.27) was the highest at month lag 4 (Table 4).

### Normalized difference vegetation index

**Fig. 3:**
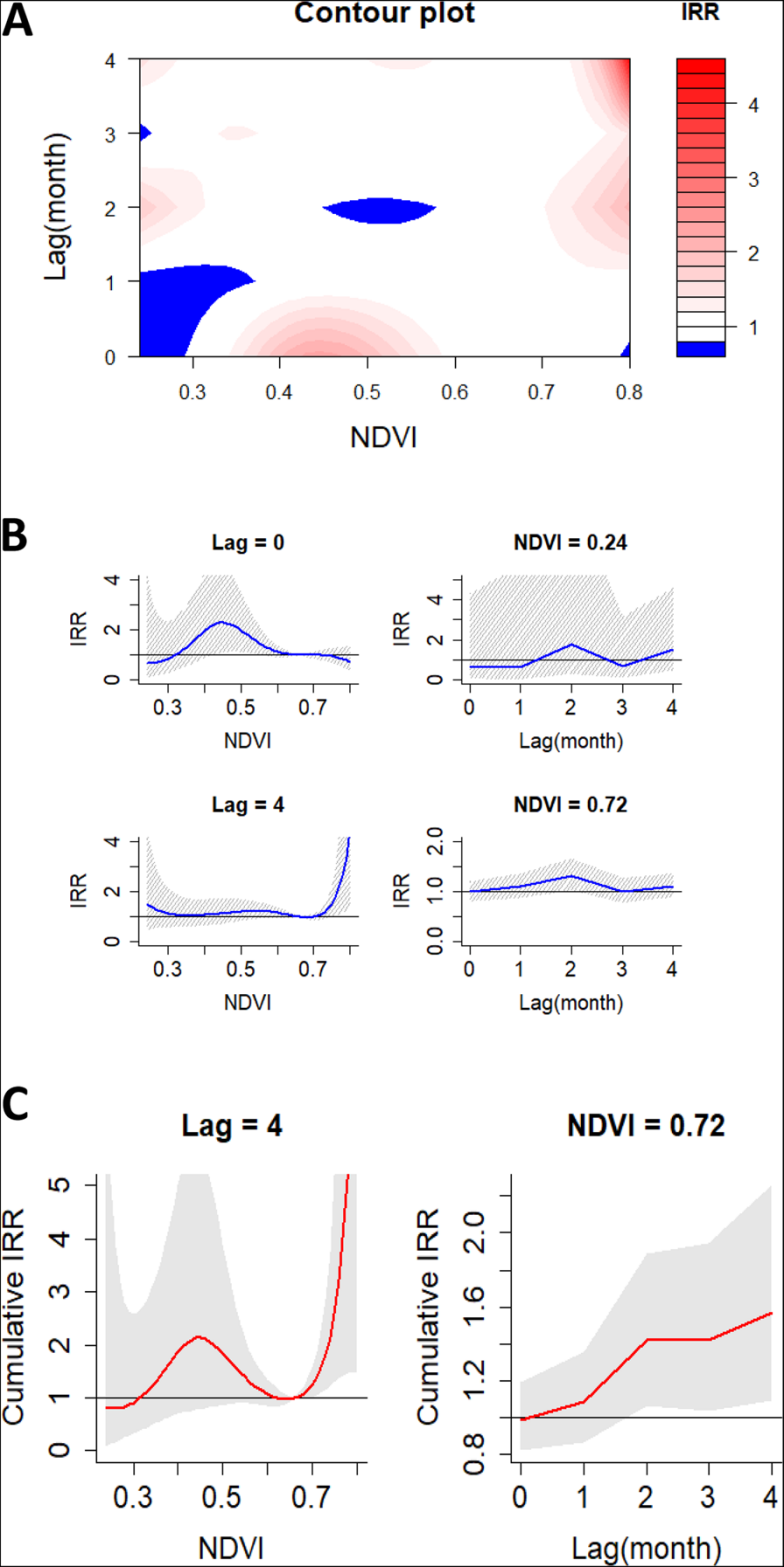
**a** Contour plots of the combined effect of time lags and normalized vegetation index (NDVI) on the incidence risk ratio of malaria. **b** Effect of specific NDVI and time lags on the incidence risk ratio of malaria. The blue lines are the mean incidence risk ratio, and the gray lines are 95% CI. **c** Effects of specific NDVI and time lags on the cumulative incidence risk ratio of malaria. The red lines are the mean incidence risk ratio, and the gray areas are 95% CI

Part a of Fig. 3 presents a summary of the non-linear relationship between monthly NDVI and malaria incidence over a four-month period. The IRR of malaria incidence increased at high NDVI values in month lags 2–4 at approximately 0.72–0.80 compared to the median observed NDVI (0.66). The separate effects of different NDVI values and 2 month lags (0 and 4 months) on the IRR together with the 95% confidence intervals are provided in part b of Fig. 3. Increase in NDVI increased the IRR drastically for values below approximately 0.5 to a peak at month lag 0. While at month lag 4, increase in NDVI reduced the IRR drastically for values below 0.3 and then increased at approximately above 0.70. Overall at low NDVI, the IRR increased to 1.80 (95% CI, 0.35–9.43) at approximately 0.24 in month lag 2 compared to the median observed NDVI (0.66). At high NDVI, the IRR increased significantly to 1.31 (95% CI, 1.04–1.65) compared to the median observed NDVI (0.66) at approximately 0.72 in month lag 2 (Table 4). The effect of NDVI on the cumulative IRR of malaria is shown in part c of Fig. 3. High NDVI increased the cumulative IRR of malaria significantly within month lags 2–4 compared to the median observed NDVI (0.66) and the IRR of 1.57 (95% CI, 1.09–2.25) was the highest at approximately 0.72 in month lag 4 (Table 4).

## Discussion

The relationship between environmental covariates and malaria burden is complex, as the effect is not only determined in the current period but may also be influenced by preceding time points. This study investigated the quantitative effect of environmental covariates on malaria incidence in high malaria transmission areas in Uganda. In these settings, temperature, rainfall and NDVI significantly affected the temporal distribution of malaria incidence. High (greater than the observed median) temperature values increased the IRR of malaria significantly in month lag 4 and the cumulative IRR at month lags 1–4 compared to the median observed temperature. Similarly, high rainfall increased the IRR of malaria significantly at the month lag 0 and the cumulative IRR at month lags 1–4 compared to the median observed rainfall. High values of NDVI increased the cumulative IRR of malaria significantly at month lags 2–4 compared to the median observed NDVI.

Malaria control remains a priority in the national health agenda, requiring planning and efficient allocation of the limited resources available [30]. Efficient allocations of resources rely not only on current measures of malaria burden but also predicting future malaria burden. Surveillance data has been used to monitor trends in malaria burden and visualization of prior seasonal peaks in different transmission settings. The addition of place of residence as part of routine surveillance data collection tool has enabled estimation of health facility catchment areas and generation of malaria incidence estimates to derive a direct measure of disease burden. Combining health facility surveillance data with environmental covariates such as rainfall, temperature and vegetation coverage available through remote-sensing sources may benefit malaria control efforts, as environmental covariates are reported to facilitate malaria transmission [31].

The relationship between environmental covariates and malaria incidence may form a strong basis for malaria early warning systems, as such prediction tools may guide planning and control of malaria outbreaks. For instance rainfall and sea surface temperature have been used for monitoring malaria early warnings in Botswana with the success of the malaria control program in reducing malaria incidence attributed to the early warnings [25]. Similarly in South Africa, prediction of malaria based on the seasonal climate forecasts showed that short-term predictions coincided closely with the observed malaria cases, which may also benefit the malaria early warning system [32]. In this study, high temperature increased the IRR of malaria at month lag 4. Knowing temperature as a key parameter in mosquito development, biting and survival with warmer temperatures increasing the infection rates as the vector reproduces faster, the likelihood of infection after a mosquito bite is amplified [33]. Even if the specific effect of temperature on the IRR of malaria increased in month lag 2, the cumulative IRR increased significantly at month lags 1–4. The increased cumulative IRR could possibly be explained by the increased multiplication rate presented by global warming increasing the length of mosquito breeding season [33]. The month lagged effects of temperature would avail time long enough to design interventions to interrupt malaria transmission, despite temperature values used in the current study being high as compared to the optimal temperature for malaria transmission of 29 °C [34]. However, this finding was consistent with previous studies which have demonstrated how temporal disease risk shifts in response to temperature changes and increase in maximum temperature increases the incidence rate of malaria significantly of the current month and later [35–37].

The current study also found high values of rainfall to significantly increase the IRR of malaria at month lag 0 in these settings. Comparable to the specific rainfall effect, the cumulative IRR of malaria was increased significantly at month lag 1–4 at approximately 200 mm. Rainfall provides avenues that facilitate mosquito breeding suggesting that these areas retain water after rains presenting suitable places for mosquito fertilization and increasing the risk of malaria infections and transmission. Although not all mosquitoes need stagnant water, they require at least some form of water to hatch eggs increasing the risk in preceding time points. The preceding time points’ malaria IRR is increased by the transcended adult mosquitoes. This finding was consistent with earlier studies. For instance a study conducted in Kenya showed positive associations between rainfall and malaria burden at lags of 2 to 4 months at rainfall approximately 100–200 mm in both lowland and highland [38].

This study also found a significant increased cumulative IRR of malaria at month lags 2–4 for high values of NDVI approximately 0.72 indicating dense vegetation. Vegetation around household residences may serve as refuge for outdoor resting of mosquitoes [39]. Conversely, sparse vegetation may limit the biting rates reducing the likelihood of malaria transmission. Deforestation which is an indicator of low vegetation cover has been shown to reduce malaria transmission significantly [40]. This study was implemented in Amazon basin which is well known to be drained by the Amazon River and its tributaries. The possible explanation is that in the current month, deforestation reduces the outdoor resting places for mosquitoes driving the mosquitoes away reducing the risk of malaria burden. Contrary with a study conducted in Kenya that have demonstrated the associations between NDVI values of 0.35 and malaria burden, the current study used 0.24 which are all in the same range of 0.2–0.5 and did not realize any specific effect significant association at any month lags [41, 42]. The possible explanation could be the difference in the transmission intensities between the current study and the former study in Kenya. The current study only considered high transmission settings while the former study compared lowlands and highlands. Ofnote highlands are prone to low mosquito population as the conditions are not friendly resulting to low infection rates.

In the present study, despite the increasing cumulative IRR for high values of environmental covariates in month lags 0–4, the rate of increase in the cumulative IRR was more in month lags 1–2 as compared to 3–4. For instance the cumulative IRR more than doubled in month lags 1–3 as compared to month lags 3–4 at temperature approximately 35 °C, more than doubled in month lags 0–2 as compared to 2–4 at rainfall approximately 200 mm, and more than doubled in month lags 0–2 as compared to 2–4 at NDVI of approximately 0.72. This may be well explained by the saturation effect, as when environmental conditions are sufficient for mosquito cycle completion, any additional value of the covariates may have little impact on the development of mosquito or parasite. This seems to suggest that interventions may be more effective if implemented in the earliest time as much as possible in order to interrupt the mosquito cycle supporting the current World Health Organization recommendations on early accurate diagnosis and treatment of malaria [43]. The current study had practical implications as the advance warnings of approaching situations advantageous to malaria epidemics will afford national malaria control programmes the freedom needed to stock commodities required to deal with impending surges or epidemics.

This study has several limitations. First, as this study was a population level study which involved environmental covariates and malaria, it is possible that some confounders may not have been considered which may have influenced the results such as socio-economic and community practices [44, 45]. Second, the data available was limited to a 24-month period, as data from previous years was only health facility cases of malaria rather than incidence as catchment areas were not available. This limited the ability to control for long-term trends. Such long-term trends in rainfall have been shown to influence malaria burden [46]. Third, this study was unable to encompass the entirety of environmental covariates, for instance because altitude did not vary over time, it was not considered as a covariate in this analysis. However, adding a health facility random variable in the model catered for the variability that was site-specific. Fourth, the study was conducted around health facilities whose data is prone to missingness may have influenced the result. Health facilities with less than 5% missing data on the village of residence for each month were included. Finally, the current study explored the associations between environmental covariates with malaria incidence in high transmission settings and the identified month-lag time points may only be applicable and generalizable to these settings. Therefore, the data should be interpreted with caution. For instance a study conducted in China showed that minimum temperature had a longer lag ranges and larger correlation coefficients for hot weather counties compared to cold weather counties. While maximum temperature was only associated with malaria cases at early lags [47].

## Conclusion

In the present study, high temperature increased the cumulative IRR of malaria significantly at month lags 1–4 compared to observed median of 30 °C. High rainfall increased the IRR of malaria significantly at month lag 0 and cummulative IRR at month lags 1–4 compared to the observed median of 133 mm. High NDVI increased the cumulative IRR significantly at month lags 2–4 compared to the observed median of 0.66. The results highlight the relevance of incorporating the effects of environmental covariates in predicting malaria when developing early warning systems. These identified complex associations are useful for designing accurate strategies for early warning, prevention, and control of seasonal malaria epidemics.

## Supplementary Information

**Additional file 1: Fig. S1.** Map of Uganda showing the study districts and malaria reference centres.

**Additional file 2: Fig. S2. a**. Estimated incidence risk ratios (IRR) for malaria as a function of temperature obtained from distributed lag models, over 1000 simulations. Estimates from the same simulation run are connected with gray lines. The red thick line represents the IRRs observed in the real dataset. Results are presented for temperatures 26 °C, 35 °C, 45 °C, taking 30 °C as a reference. The results were obtained when simulating data with the following IRRs: At temperature 26 °C: IRR = 1 at lags 0 and 2, IRR = 1.31 at lag 1, IRR = 1.27 at lag 3, and IRR = 1.24 at lag 4; at temperature 35 °C: IRR = 1.28 for lags 0, IRR = 1.45 for lags 1, IRR = 1.24 for lags 2, IRR = 1.70 at lag 3, IRR = 1.24 at lag 4; at temperature 45 °C: IRR =1.29 at lag 0, IRR = 1 for lags 1 and lag 4, IRR = 1.07 at lag 2, IRR =1.48 at lag 3. **b**. Estimated IRR for malaria as a function of rainfall obtained from distributed lag models, over 1000 simulations. Estimates from the same simulation run are connected with gray lines. The red thick line represents the RRs observed in the real dataset. Results are presented for rainfall 3 mm, 200 mm, and 247 mm, taking 133 mm as a reference. The results were obtained when simulating data with the following IRRs: At rainfall 3 mm: IRR = 1 at lags 0,2 and 3, IRR = 1.15 at lag 1, and IRR = 1.60 at lag 4; at rainfall 200 mm: IRR = 1.12 for lags 0, IRR = 1 at lags 1,3 and 4, IRR = 1.03 at lag 2; at rainfall 247 mm: IRR =1 at lag 0, IRR = 2.13 at lag 1, IRR = 1.46 at lag 2, IRR = 1.95 at lag 3, IRR = 2.20 at lag 4. **c**. Estimated IRR for malaria as a function of NDVI obtained from distributed lag models, over 1000 simulations. Estimates from the same simulation run are connected with gray lines. The red thick line represents the RRs observed in the real dataset. Results are presented for NDVI values of 0.24, 0,50,0.72, taking 0.66 as a reference. The results were obtained when simulating data with the following IRRs: At NDVI 0.24: IRR = 1 at lags 0 and 1, IRR = 1.04 at lag 2,IRR = 1.98 at alg3, and IRR = 1.37 at lag 4; at NDVI 0.50: IRR = 1.13 for lags 0, IRR = 1.01 at lag 1, IRR = 1 at lag 2,IRR = 1.22 at lag 3, and IRR = 1.34 at lag 4; at NDVI 0.72: IRR =1.17 at lag 0, IRR = 1 at lag 1 and 3, IRR 1.12 at lag 2, IRR = 1.07 at lag 4.

**Additional file 3: Fig. S3.** Temporal changes in monthly malaria incidence over the 24-month observation period around each MRC and all-sites combined.

## Data Availability

The datasets used for this study are available from the corresponding author on reasonable request.

## Abbreviations

CI: Confidence Intervals
CHIRPS: Climate Hazards Group InfraRed Precipitation with Station data
DLNM: Distributed lag nonlinear models
IRR: Incidence Rate Ratio
MODIS: Moderate Resolution Imaging Spectroradiometer
MRCs: Malaria Reference Centres
NMCD: National Malaria Control Division
NDVI: Normalized Difference Vegetation Index
PY: Person Years
QAIC: Quasi Akaike Information Criterion
QGIS: Quatuam Geographical Information System
REC: Research Ethics Committee
UMSP: Uganda Malaria Surveillance Project
WHO: World Health Organization
