# Supplementary figures and images for: Associations between environmental covariates and temporal changes in malaria incidence in high transmission settings of Uganda: a distributed lag nonlinear analysis
Source: BMC Public Health. 2021 Oct 30;21:1962. doi: 10.1186/s12889-021-11949-5 (PMC8557030; doi:10.1186/s12889-021-11949-5)

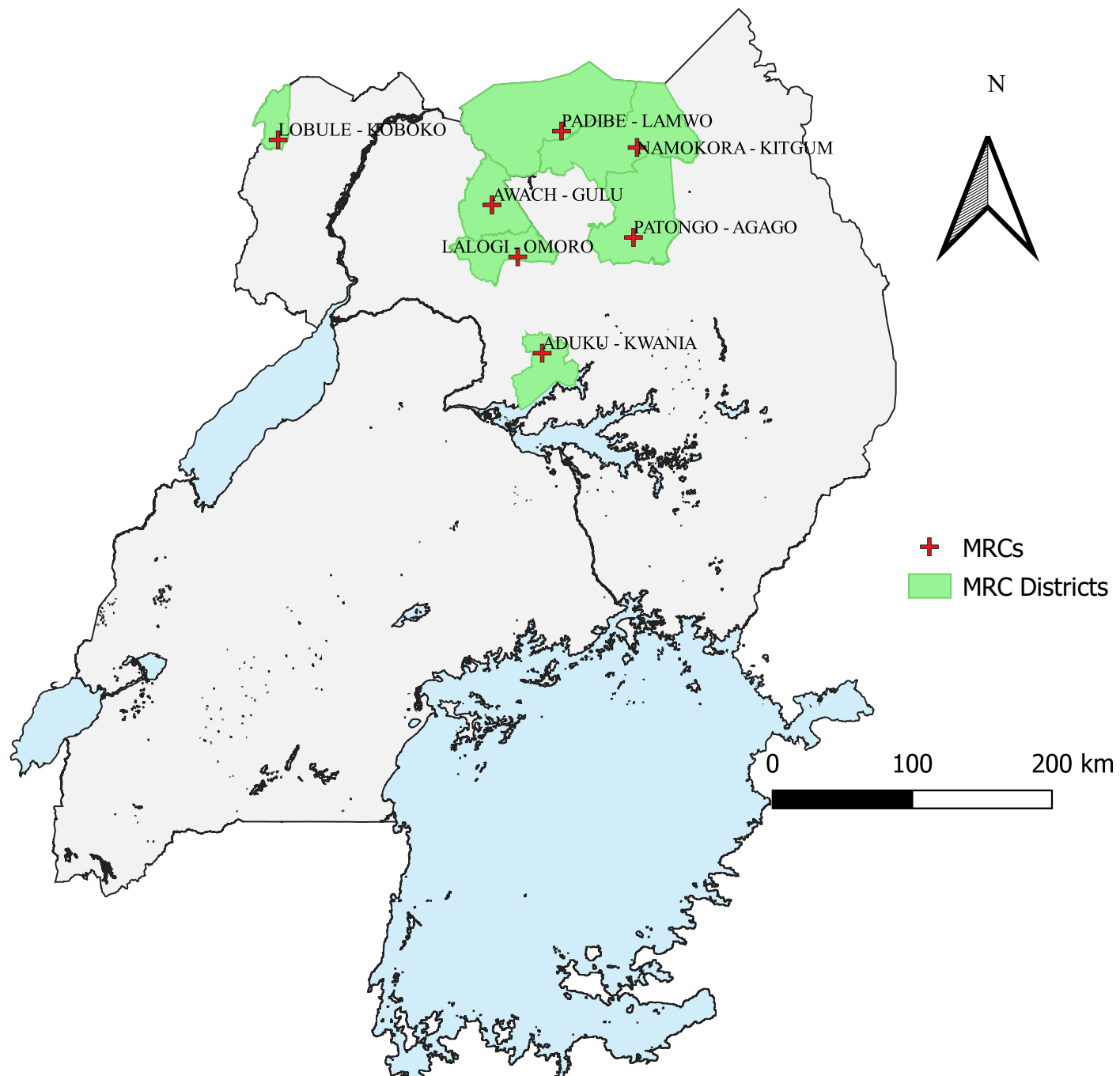

Supplement: Supplementary file 1 — Additional file 1: Fig. S1. Map of Uganda showing the study districts and malaria reference centres. [file 12889_2021_11949_MOESM1_ESM.pdf]

A

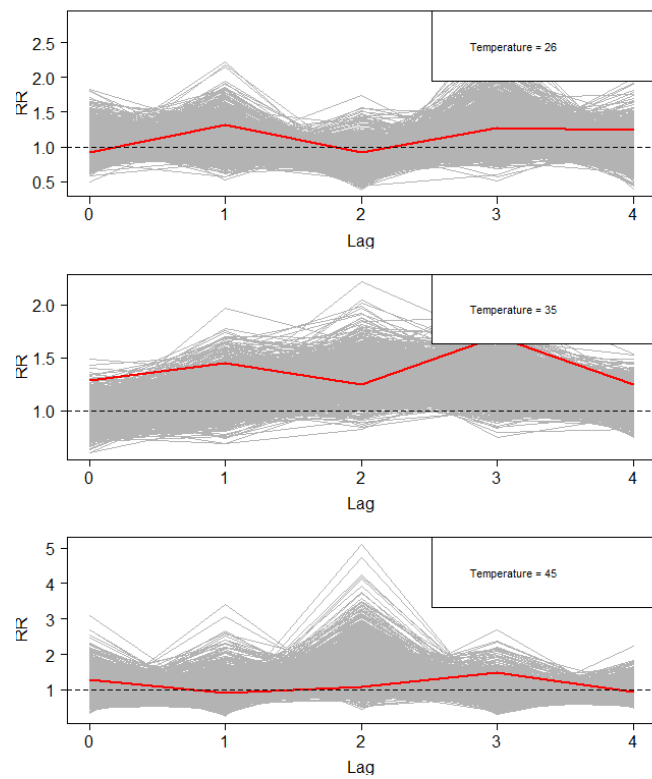

B

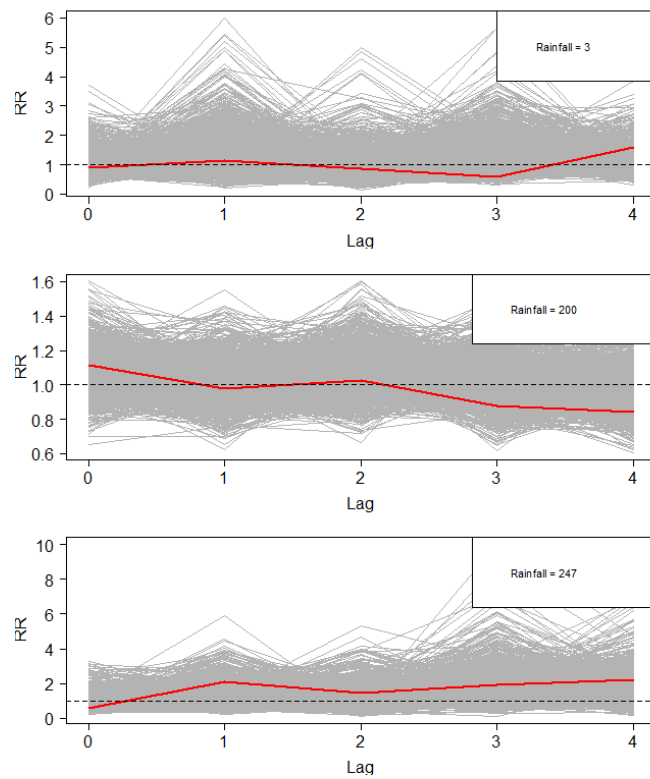

C

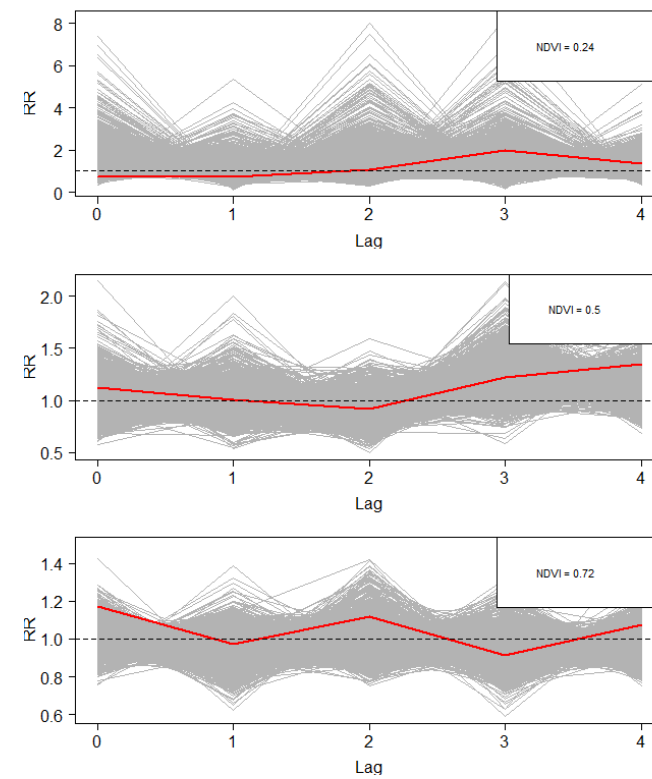

RR=Incidence risk ratio

Supplement: Supplementary file 2 — Additional file 2: Fig. S2. a. Estimated incidence risk ratios (IRR) for malaria as a function of temperature obtained from distributed lag models, over 1000 simulations. Estimates from the same simulation run are connected with gray lines. The red thick line represents the IRRs observed in the real dataset. Results are presented for temperatures 26 °C, 35 °C, 45 °C, taking 30 °C as a reference. The results were obtained when simulating data with the following IRRs: At temperature 26 °C: IRR = 1 at lags 0 and 2, IRR = 1.31 at lag 1, IRR = 1.27 at lag 3, and IRR = 1.24 at lag 4; at temperature 35 °C: IRR = 1.28 for lags 0, IRR = 1.45 for lags 1, IRR = 1.24 for lags 2, IRR = 1.70 at lag 3, IRR = 1.24 at lag 4; at temperature 45 °C: IRR =1.29 at lag 0, IRR = 1 for lags 1 and lag 4, IRR = 1.07 at lag 2, IRR =1.48 at lag 3. b. Estimated IRR for malaria as a function of rainfall obtained from distributed lag models, over 1000 simulations. Estimates from the same simulation run are connected with gray lines. The red thick line represents the RRs observed in the real dataset. Results are presented for rainfall 3 mm, 200 mm, and 247 mm, taking 133 mm as a reference. The results were obtained when simulating data with the following IRRs: At rainfall 3 mm: IRR = 1 at lags 0,2 and 3, IRR = 1.15 at lag 1, and IRR = 1.60 at lag 4; at rainfall 200 mm: IRR = 1.12 for lags 0, IRR = 1 at lags 1,3 and 4, IRR = 1.03 at lag 2; at rainfall 247 mm: IRR =1 at lag 0, IRR = 2.13 at lag 1, IRR = 1.46 at lag 2, IRR = 1.95 at lag 3, IRR = 2.20 at lag 4. c. Estimated IRR for malaria as a function of NDVI obtained from distributed lag models, over 1000 simulations. Estimates from the same simulation run are connected with gray lines. The red thick line represents the RRs observed in the real dataset. Results are presented for NDVI values of 0.24, 0,50,0.72, taking 0.66 as a reference. The results were obtained when simulating data with the following IRRs: At NDVI 0.24: IRR = 1 at lags 0 and 1, IR [file 12889_2021_11949_MOESM2_ESM.pdf]

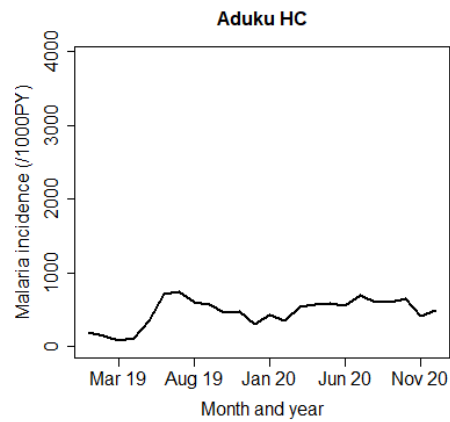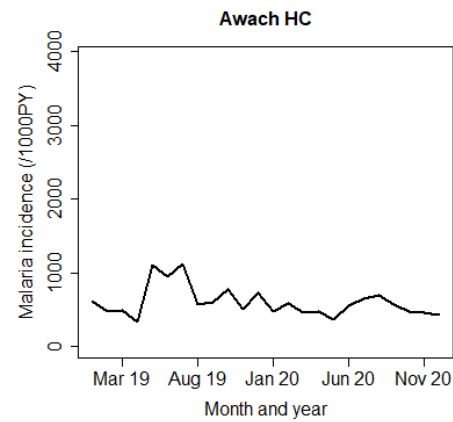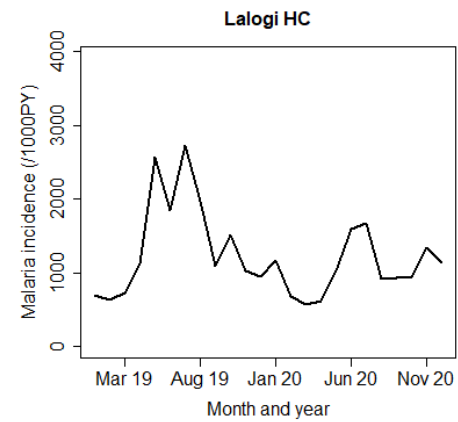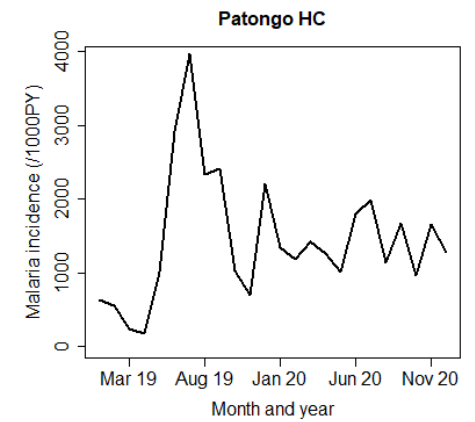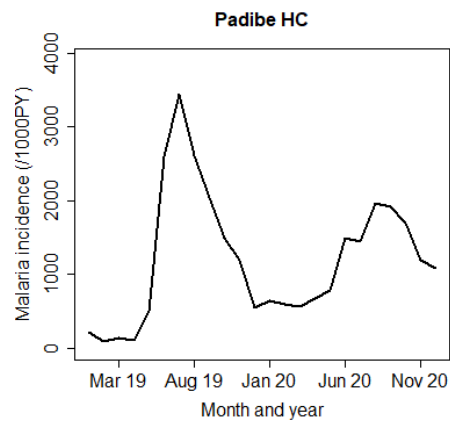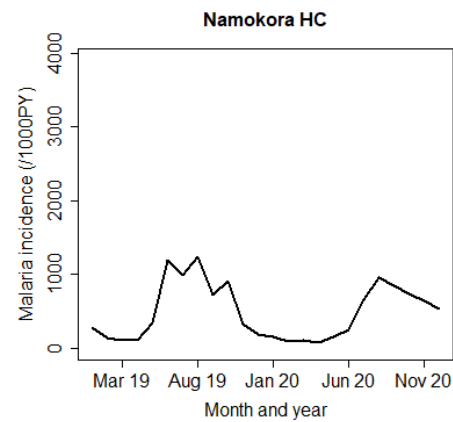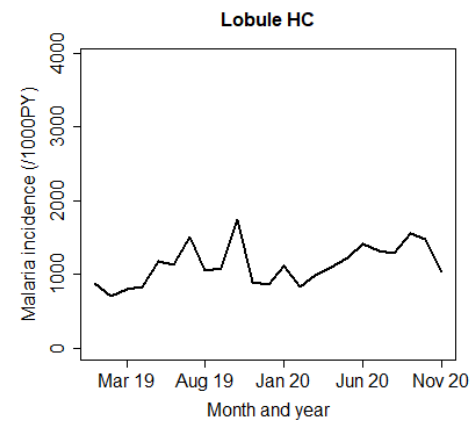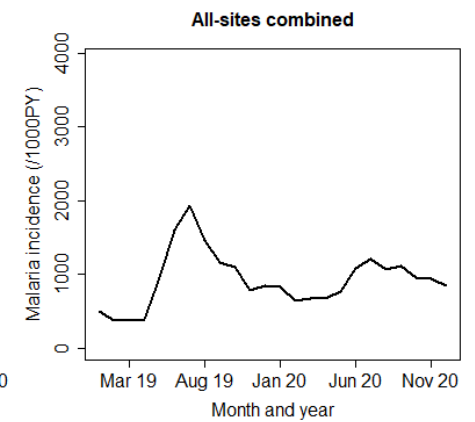

Supplement: Supplementary file 3 — Additional file 3: Fig. S3. Temporal changes in monthly malaria incidence over the 24-month observation period around each MRC and all-sites combined. [file 12889_2021_11949_MOESM3_ESM.pdf]
